# Development and formative evaluation of an innovative mHealth intervention for improving coverage of community-based maternal, newborn and child health services in rural areas of India

**DOI:** 10.3402/gha.v8.26769

**Published:** 2015-02-16

**Authors:** Dhiren Modi, Ravi Gopalan, Shobha Shah, Sethuraman Venkatraman, Gayatri Desai, Shrey Desai, Pankaj Shah

**Affiliations:** 1 Community Health Department, SEWA Rural, Bharuch, Gujarat, India; 2 Argusoft India Ltd., Gandhinagar, Gujarat, India

**Keywords:** mHealth, ASHAs, acceptability, uptake, implementation research, complex intervention

## Abstract

**Background:**

A new cadre of village-based frontline health workers, called Accredited Social Health Activists (ASHAs), was created in India. However, coverage of selected community-based maternal, newborn and child health (MNCH) services remains low.

**Objective:**

This article describes the process of development and formative evaluation of a complex mHealth intervention (ImTeCHO) to increase the coverage of proven MNCH services in rural India by improving the performance of ASHAs.

**Design:**

The Medical Research Council (MRC) framework for developing complex interventions was used. Gaps were identified in the usual care provided by ASHAs, based on a literature search, and SEWA Rural's^1^ three decades of grassroots experience. The components of the intervention (mHealth strategies) were designed to overcome the gaps in care. The intervention, in the form of the ImTeCHO mobile phone and web application, along with the delivery model, was developed to incorporate these mHealth strategies. The intervention was piloted through 45 ASHAs among 45 villages in Gujarat (population: 45,000) over 7 months in 2013 to assess the acceptability, feasibility, and usefulness of the intervention and to identify barriers to its delivery.

**Results:**

Inadequate supervision and support to ASHAs were noted as a gap in usual care, resulting in low coverage of selected MNCH services and care received by complicated cases. Therefore, the ImTeCHO application was developed to integrate mHealth strategies in the form of job aid to ASHAs to assist with scheduling, behavior change communication, diagnosis, and patient management, along with supervision and support of ASHAs. During the pilot, the intervention and its delivery were found to be largely acceptable, feasible, and useful. A few changes were made to the intervention and its delivery, including 1) a new helpline for ASHAs, 2) further simplification of processes within the ImTeCHO incentive management system and 3) additional web-based features for enhancing value and supervision of Primary Health Center (PHC) staff.

**Conclusions:**

The effectiveness of the improved ImTeCHO intervention will be now tested through a cluster randomized trial.

To facilitate delivery of proven community-based maternal, newborn and child health (MNCH) services, a new cadre of village-based community health workers, called Accredited Social Health Activist (ASHA), was created in 2005 under the aegis of the National Rural Health Mission in India (1). Each ASHA is a native village woman who has the equivalent of at least a 10th-grade education. There is one ASHA for every 1,000 people in the countryside, and there were 863,503 ASHAs in India in 2013 (2). ASHAs are expected to contribute 3–5 h of service each day (3, 4). Many of the community-based MNCH services are expected to be implemented or facilitated by ASHAs during their home visits to beneficiaries (3). Evaluations of the ASHA program have found that ASHAs seem to perform well in mobilizing beneficiaries for uptake of health services such as institutional delivery and immunization. However, some evaluations have noted that coverage of other selected MNCH services delivered by ASHAs is low and that the institutional capacity for providing support and supervision must be improved in order to realize the huge inherent potential of the ASHA program (1, 2, 5, 6).

This article describes the process of development and formative evaluation of a complex intervention (called ImTeCHO) based on mobile phone technology (mHealth) for improving the performance of ASHAs and overcoming implementation bottlenecks to improve coverage of selected proven MNCH services. A complex intervention is characterized as having a number of components that are interconnected (7). We used the framework prescribed by the Medical Research Council (MRC), United Kingdom, for the development and evaluation of complex interventions, along with the documented experiences of other researchers involved in designing complex services elsewhere in India (7, 8). The MRC framework suggests the following phases: 1) identify gaps in usual care, 2) design components of intervention and model their impact, 3) evaluate acceptability and feasibility of components, and 4) pilot operational delivery of the intervention (7, 8).

## Intervention development

### Phase 1: identifying gaps in usual care

Gaps in the usual care to be provided or facilitated by ASHAs along with underlying implementation bottlenecks responsible for the gaps were identified through a literature search and SEWA Rural's three decades of experience in implementing several community-based interventions. SEWA Rural is a voluntary organization working towards the overall development of the tribal community of south Gujarat, India, since 1980. Following are the findings of the literature review.

#### Gap 1: low coverage of selected MNCH services to be delivered or facilitated by ASHAs (1, 5)

Some of the critical proven MNCH services to be delivered by ASHAs include the following: 1) mobilizing pregnant women for antenatal examination, 2) motivating pregnant women for delivery in hospital through preparation of a birth plan, 3) escorting pregnant women to the hospital for delivery, 4) home-based newborn care, 5) motivating mothers to breast-feed exclusively for the first 6 months, and 6) mobilizing children for immunization (3).

The performance of ASHAs on some of these tasks was below the predicted success rate. For example, the coverage (national average) of selected services among postpartum women reported in one ASHA evaluation was as follows: at least three antenatal care visits (58%), postpartum-care counseling (34%), visit on the day of birth (34%), assistance with early breastfeeding (28%), and identification of sick newborns (39%) (5). The coverage of interventions is even worse for tribal areas and certain states (5, 9, 10) . Some of the potential implementation bottlenecks causing this suboptimal performance of ASHAs might be inadequate training leading to poor skills, unsatisfactory supervision and support, and lack of understanding about the role of ASHAs at various levels of the health system (1, 5).

#### Gap 2: low coverage of care among complicated maternal, newborn and child cases (2, 6)

ASHAs are expected to screen pregnant women, newborn babies, and young children to identify common complications, to refer cases with serious complications to the hospital, and to manage less serious complications at home (11). However, only 60% of women with any antenatal or postnatal complications sought and received care in Gujarat in 2007 (9). In a study conducted at Wardha, 46% of newborns with complications did not get any care (12). A large proportion of pregnant women, neonates, and children with complications do not seek and receive care at a health facility (8, 13–15). Large numbers of complicated cases who stay home do not receive any care (13). The reasons for this might be the presence of various barriers for seeking care, such as insufficient advanced planning and knowledge about danger signs (12, 13, 16). Case management of common newborn and childhood complications by ASHAs is one of the important functions envisioned in the ASHA program; however, their performance in this regard is highly variable across the states and clarity regarding their role among administrators of health programs is inadequate (5). Inadequate training, skills, and support make it difficult for ASHAs to identify, triage, and manage complicated cases that are unable to go to a health facility (1, 6). In addition, ASHA supervisors (described below) do not receive real-time information about these cases, although they are health-care providers qualified to manage complications.

#### Gap 3: inadequate supervision and support of ASHAs (1, 5, 6)

ASHAs are to be supported and supervised by ASHA facilitators and Auxiliary Nurse Midwives (ANMs) of the Primary Health Center (PHC) through the replenishment of supplies, regular payment of performance-based incentives, supervision during field visits, and mentoring (3). The PHC is operational and the administrative unit for all villages covered by it; each PHC covers approximately 20,000 to 30,000 people. Each PHC has a team of two medical officers (doctors), four to six ANMs, two to three ASHA facilitators, between twenty and twenty-five ASHAs, one Lady Health Visitor, and three male multipurpose workers (4). However, lack of regular and reliable supervision is noted in many studies (1). ASHA evaluations have observed that 87% of ASHAs studied reported that there was lot of delay in payment of incentives and 25% of ASHAs felt that they are not incentivized enough (1, 17). Additionally, the replenishment of supplies was often erratic (1, 5).

### Phase 2: identifying the components (mHealth strategies) of the intervention and modeling their impact

#### Identifying the components of the intervention

**Fig. 1:**
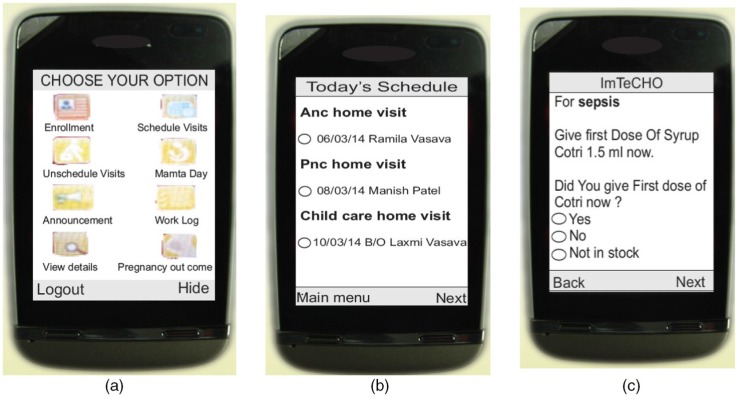
Selected screen shots of the ImTeCHO application (a) main menu, (b) ASHAs’ Daily Schedule, (c) diagnosis with management guidelines.

The components of the intervention (mHealth strategies) were identified by 1) finding potential solutions to overcome the underlying implementation bottlenecks responsible for the gaps in care identified above; 2) applying the concept of user- (human-) centered design to create solutions by focusing on tasks and the needs of end-users (here, health workers) through an iterative process; and 3) tapping into the principles of implementation science, which explores ways to implement proven interventions in real-life settings (18, 19). The underlying assumption, based on SEWA Rural's three decades of experience in community health, was that the village-based frontline workers adhere to interventions and perform well if they are adequately supported and supervised, as well as when the project objectives are aligned with their own motivations. Hence, the following components were included in the ImTeCHO mobile phone application to be used by ASHAs. The scope of the application included almost all MNCH services to be delivered at the community level through ASHAs, ANMs, and PHC staff. A brief description of implementation bottlenecks and corrective mHealth strategies is shown in Table 1. Figure 1 shows selected screen shots of the ImTeCHO application.

- Job-aid for provider scheduling: Once an ASHA registers a pregnant woman or a child through the ImTeCHO application, the *system* automatically generates a complete schedule of home visit ‘tasks’ for the ASHA. The system displays tasks on the ASHA's Daily Schedule module in the ImTeCHO application on the appropriate date. The system sends a task reminder to the ASHA a few days prior to the patient's due date, providing flexibility to the ASHA so that she can complete the assigned tasks at a time convenient to her. Such tasks include home visitations for providing antenatal care, home-based-newborn care, and child care; reporting the outcome of each pregnancy; and follow-up visits for complicated cases. The color of the text displaying the task on the mobile phone screen turns from yellow to red if the task is not completed by the due date, which indicates to the ASHA the need to prioritize that particular task. Each task has a corresponding *digital form*, which needs to be filled out by the ASHA in order to mark the task as completed.
- Job-aid for behavior change communication (BCC): For effective BCC, each digital form (described above) contains a relevant checklist and brief video clips (e.g. the home-based newborn care task would have a video about danger signs in newborns), so that the correct video and information is shown to the right beneficiary at the right time (see Supplementary files).
- Job-aid for diagnosis and patient management (electronic decision support): To ensure timely identification and management of complications, each digital form contains checklists to remind ASHAs to perform and record recommended examinations, which are linked to an algorithm. The algorithm makes it possible for the ImTeCHO application to show a probable diagnosis, color-coded risk stratification (red, yellow, or green according to the severity of complications), and a customized treatment plan once the checklists are completed.

**Table 1:** Components of the ImTeCHO intervention and how it addresses gaps in services to be provided by ASHAs and PHC staff

No.	Implementation bottlenecks responsible for gaps in services to be provided by ASHAs	Components of the ImTeCHO intervention/mHealth strategies targeting each implementation bottleneck
A. Low coverage of MNCH services to be provided or facilitated by ASHAs		
1	Poor skills of ASHAs (do not remember the algorithm, triage system, poor counseling skills, inadequate training) (5)	BCC job-aid, diagnosis and patient management job-aid: Use of checklist, images, videos (for common counseling subjects), algorithms, and automated risk stratification assist ASHAs to overcome skill deficits.
2	Difficult for ASHAs to remember services due to be provided to beneficiaries	Scheduling job-aid: Automated scheduling and reminder alerts based on recommended schedule.
3	Poor motivation due to ASHAs’ perceived inadequacy of incentives against work done (1, 5)	ASHA supervision and support management job-aid: Automatic calculation of performance-based incentives, based on digital records of services provided by ASHA on time. Audit trail and transparency to reflect amount and timeliness of incentive payment.
4	Irregular supplies (1)	ASHA supervision and support management job-aid: Supply management system that records and reports low inventory to the appropriate authority.
5	ASHAs are not recruited in some villages (vacant positions)	ASHA supervision and support management job-aid: The fact that ASHA positions are vacant is highlighted to program managers, because there is no available performance data for those particular villages.
6	Barriers to behavior change at household level (12, 13, 16)	BCC job-aid: Checklist in digital form to assess and address such barriers. Eleven mobile-based short videos, in form a 2–3 min picture story, assist ASHAs to counsel about key health practices during their home visits to beneficiaries. Each video was created using various behavior change theories.
B. Low care seeking from appropriate health personnel		
7	Difficult for ASHA to screen, identify, and triage complicated cases because of insufficient training, skill, and support (1, 6)	Diagnosis and patient management job-aid: Mobile phone equipped with algorithms that provide diagnosis and risk stratification based on information entered by ASHA.
8	Presence of various barriers (such as lack of knowledge about seriousness of morbidity, advance planning to deal with complications among beneficiaries) reduces the chances of referral of complicated cases to health facility (15, 16)	BCC job-aid: Counseling videos about danger signs is available in mobile phone to increase knowledge about complications Use of checklist to encourage households to plan for complications (complication readiness).
9	Difficult for ASHA to manage selected complicated cases at home (who refuse to get referred), especially complicated newborn and child cases, because of inadequate training, skills, and support (1)	Diagnosis and patient management job-aid: Mobile phone equipped with algorithms provides management guidelines to ASHA for selected maternal, newborn and child complications.
10	ANMs and medical officers do not know in real time about mothers and newborns with complications	Diagnosis and patient management job-aid: Automated alert and SMS goes to an ANM and medical officer instantly if a complicated case is identified by ASHA during home visit; thus, the ANM can plan to visit such cases in near future.
C. Inadequate supervision and support to ASHAs		
11	Inadequate authentic information about ASHA's performance (2)	ASHA supervision and support management job-aid: Real-time and user friendly information regarding performance of ASHAs is available through the ImTeCHO web interface.
12	Difficult to verify the truthfulness of monthly reports provided by ASHAs regarding their performance	ASHA supervision and support management job-aid: A time-stamp and information about the duration of visit are available for every home visit made by ASHAs. Additionally, the application prompts ASHAs to take a photo of beneficiary's health card or house for each home visit. Such features help program manager to assess truthfulness of data.
13	Effort-intensive process of managing supplies, along with calculating and disbursing incentives to ASHA	ASHA supervision and support management job-aid: System seeks information about remaining supplies from each ASHA every month and sends low inventory information to PHC staff. Electronic record of ASHA's performance and automated calculation of incentives reduce efforts required by support staff at PHC.
14	Lack of real-time information about complicated cases to ANMs and medical officers	Diagnosis and patient management job-aid: List of complicated cases is available on web interface as soon as complicated case is diagnosed by ASHA.

The web interface to be used by medical officers and PHC staff was prepared based on the following components:

- Job-aid for supervision and support of ASHAs: The ImTeCHO web interface provides real-time information and tools for medical officers and PHC staff for providing timely support (e.g. tracking high risk patients, alerts for low supply inventory and deaths, human resource management, supply chain management, automatic calculation of performance-based incentives and motivation, electronic health records, vital events tracking) and supervision of ASHAs. Tools to improve supervision include real-time information about the performance of ASHAs in the form of process indicators and coverage of proven MNCH services.

The mobile phone and web application was developed on the Java platform. The ImTeCHO mobile phone application is powered by the *mAID* platform, created by Argusoft India Ltd., the information technology partner for this project. The ImTeCHO application was customized for SEWA Rural and further extended with collaborative domain knowledge inputs from SEWA Rural experts. The system was built on modular architecture with well-defined interfaces that are extensible and interoperable with other systems for data exchange. Strict security measures were put in place, including encryption of data along with use of username and password. The database server and web application were installed on a Tier-1 high-security data center. The application has full Unicode Transformation Format compatibility and can support any Indian language. The content of the application (text, videos, etc.) can be modified from the web interface. Its open source code makes it possible to adapt it in future, as required.

**Fig. 2:**
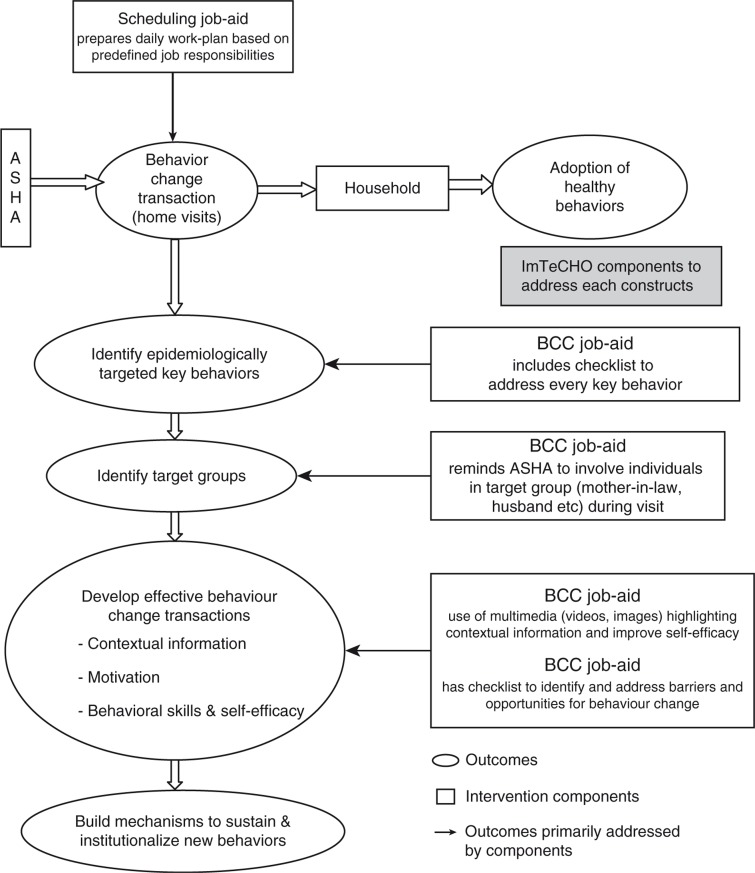
Modeling of the intervention components for increasing coverage of MNCH services by ASHAs using mobile phones (ImTeCHO) as job-aid. Adapted from Ref. (20).

**Fig. 3:**
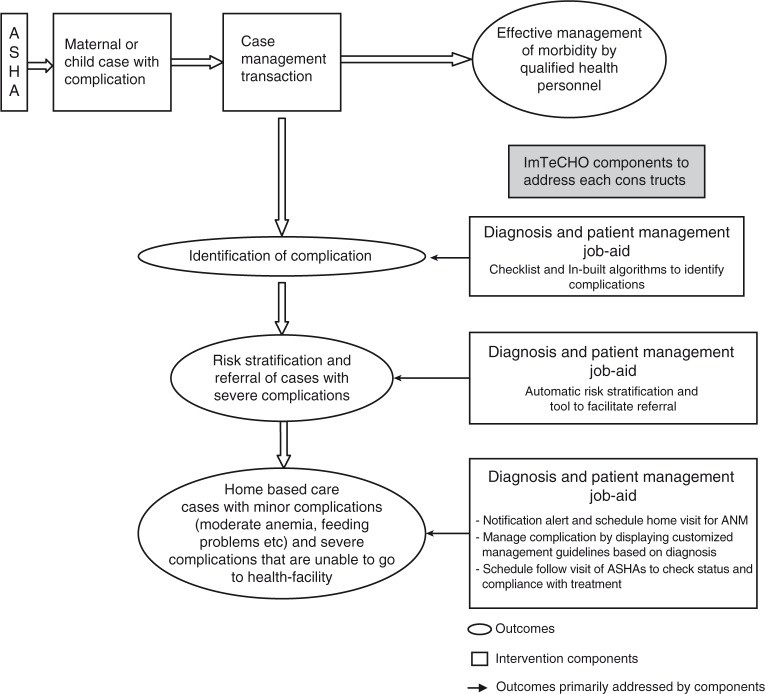
Modeling of the intervention components for increasing coverage of care of maternal and newborn cases with complications using mobile phones as job-aid.

Modeling of the components of the intervention to estimate its impact on outcomes is shown in Figs. 2 and 3.

#### Delivery of the intervention

It was envisioned that existing PHC staff would deliver the intervention, with SEWA Rural staff having a facilitative role, which was defined a priori; this was done to assess the generalizability and potential scale-up in larger areas that may not have the strong presence of SEWA Rural. The following individuals would deliver the intervention.

All ASHAs were provided, free of cost, with a mobile smartphone (Nokia ASHA 311), which came with the ImTeCHO application installed, an Internet plan costing Rs 150 (US$2.50) per month per ASHA (paid monthly by SEWA Rural), and talk time worth Rs 60 (US$1) for every 6 months. ASHAs were expected to log in to the ImTeCHO application every day except Sundays to 1) review their schedule for that day and complete assigned tasks on time; 2) enroll new beneficiaries; and 3) report information about deaths, emergencies, migrations, referrals, and services provided at the monthly Village Health and Nutrition Day (VHND). The data was transmitted using the General Packet Radio Service (GPRS) network to a server, where it was stored. If GPRS was not available, the data was stored in the mobile phone and sent to the server when GPRS became available. Every year, the clients of the average ASHA included 10–15 pregnant women, 10–15 newborn babies, and 20–30 children under the age of 2 years, considering an average population of 1,000 per ASHA. ASHAs received additional incentives (on average Rs 400, US$6) for participating in the ImTeCHO project and for carrying out recommended tasks that are not incentivized through that ASHA program (e.g. home visits to meet pregnant women and young children). The medical officers at PHC were expected to log in to the ImTeCHO web interface to supervise and support the ASHAs; most of the PHCs in Gujarat already had computers with Internet access. Selected performance and health indicators were monitored by higher-level government health officials (block, district, and state levels) through a dashboard on the ImTeCHO web interface.

Various housekeeping functions were identified for optimal delivery of this technology-intensive intervention, including troubleshooting the mobile phone application and web interface, assisting users in utilizing data to facilitate their responsibilities, and identifying any ASHAs who might not be adhering to the intervention by tracking innovative process indicators. Some of the selected process indicators that were tracked included login rate (proportion of days ASHAs logged in to the ImTeCHO mobile phone application to total number of days), task completion rate (proportion of scheduled tasks completed), duration of home visits (an indirect measure of the quality of a home visit), and time elapsed between date of delivery and the first home visit by ASHAs. These process indicators were important tools for monitoring adherence to the intervention. These housekeeping functions were carried out by a new cadre of worker, namely an ImTeCHO facilitator, who was a SEWA Rural employee. The Emergency Response Team (ERT), composed of SEWA Rural staff, assisted the ASHAs with patient care in severely complicated cases who were unable to get referred to a health facility. Argusoft India Ltd. provided support as required for maintenance and modification of the application.

We anticipated resistance among the ASHAs and PHC staff towards use of ImTeCHO because it involved a significant change in their working environment as a result of the use of technology; hence, *change management* was an important part of delivery of the intervention (21). Change management included regularly communicating the benefits of using ImTeCHO to health workers, linking monetary incentives to performance recorded through the application, channeling formal instructions from higher level health officials through the application, and involving health workers in refining the ImTeCHO through an iterative process (see Supplementary files).

The intervention was rolled out in three stages. A series of checklists was used and validated to guide its delivery and create a clear, scalable implementation plan for subsequent trial and roll-out:

Stage 1: preparations before training and implementation

This stage included mapping the intervention area for GPRS connectivity, obtaining information on all health workers to create their user profiles in the ImTeCHO system, orienting all PHC staff along with community leaders about ImTeCHO, procuring mobile phones with SIM cards, getting the mobile phones ready (e.g. downloading the application, videos, installing vernacular fonts), and preparing the training site (e.g. mobile connectivity and planning logistics such as meals, lodging).

Stage 2: training and on-the-ground mentoring of ASHAs and PHC staff

ASHAs received a 3-day-long refresher course that focused on improving the knowledge and skills of ASHAs related to caring for pregnant woman, newborn babies, and children. This course was followed by 5 days of training in use of the ImTeCHO mobile phone application; classroom training was followed by on-the-ground mentoring and certification by trained mentors from SEWA Rural. A separate training application was used for hands-on practice during this period. Subsequently, PHC staff received 2 days of training in use of the ImTeCHO web application.

Stage 3: stabilization and maintenance of delivery of intervention

ASHAs and PHC staff used the ImTeCHO mobile and web application during their routine daily activities, assisted by the ImTeCHO facilitator. The ERT visited and managed all high-risk cases identified by ASHAs through the ImTeCHO application. All other SEWA Rural staff withdrew from delivery of the intervention.

### Phase 3: evaluating acceptability, feasibility, and usefulness of components

This evaluation was accomplished by interviewing potential beneficiaries (pregnant women and mothers of infants), ASHAs, ASHA facilitators, medical officers, and district- and state-level policy makers and administrators over the course of few months. Various components were described and simulated with help of mock simulation software. The goals of this phase were 1) to get suggestions from potential users of ImTeCHO in order to refine the content of various components; 2) to learn about past experiences in implementation of existing ASHA programs in order to refine the goals and content of intervention; and 3) to assess the usefulness, acceptability, feasibility, and underlying assumptions of the components and delivery mechanisms.

Some of the findings of the above process were as follows:

- It was abstract for health workers to imagine the various components of the intervention because of their lack of exposure to mobile phone and web applications; hence, it was difficult to get their feedback.
- The component related to support should include incentive management, considering the ASHAs’ perceived irregularities with payment. The PHC support staff wanted to have information about incentive payments, which might be needed in case of an audit.
- Many ASHAs had inadequate skills (for example, thermometer use) as service providers and needed to be retrained to get up-to-date. PHC staff and ASHAs will need to be educated about the role of ASHAs as a service provider (for common maternal and childhood complications), instead of only as a mobilizer for various health services.
- None of the ASHAs had a smartphone; hence, phones will need to be provided to them. ASHAs preferred to have a mobile with dual-SIM so that they can use the same phone for personal use with a personal SIM. Few ASHAs were concerned about unreliable mobile phone signals and electricity supply in their villages, further motivating us to develop the ImTeCHO application, which could be used off-line in such areas.
- Medical officers wanted to further strengthen components related to supervision of ANMs and ASHAs by having a line listing beneficiaries who are overdue for selected services. Higher-level officials requested that selected key indicators be made available through the web application so that they can supervise medical officers.
- Almost all potential users appeared apprehensive about the impact of the ImTeCHO on their work load, remuneration, and supervision. Change management and constant efforts to keep ASHAs motivated appeared to be critical for effective delivery of the intervention.
- Users might need additional technology and related support. Such support (e.g. training all users to use the application, troubleshooting technology-related issues, and monitoring to ensure adherence to intervention) is essential for initiating and maintaining technology-intensive interventions, such as ImTeCHO. These observations reinforced our plans to provide active facilitation through SEWA Rural staff.
- None of the potential beneficiaries objected to having their photo taken with a mobile phone, which was required to ensure that ASHAs had actually visited them. Showing BCC videos through mobile phones would not be culturally inappropriate in rural areas as per the potential beneficiaries.

### Phase 4: piloting operational delivery of the intervention

The objectives of this phase of piloting were 1) to assess acceptability, usefulness, feasibility, and user-friendliness and to test the underlying assumptions of all components of the intervention; 2) to identify challenges and barriers for delivering the intervention, including training of users; and 3) to study tools for monitoring implementation of the intervention. A detailed report on formative evaluation will be published as a separate publication. Below, a brief summary is shared.

The intervention was piloted through all 45 ASHAs and the staff of two PHCs, serving 45 villages (population: 45,000) of Jhagadia block, Gujarat, between August 2013 and February 2014. Gujarat is located in western India; its population was 60 million in 2011 and per capita annual income was Rs 22,553 (US$450) (22, 23). These two PHCs were selected because of their proximity to SEWA Rural's main campus, the good mix of remote and semiurban population in the area, and the long-time presence here of SEWA Rural's service delivery programs. This was a rural, predominantly tribal area consisting largely of marginal farmers and landless laborers, with more than one-third of the population living below the poverty line. In 2011, Jhagadia block's maternal mortality ratio was 161 deaths/100,000 live births, whereas its neonatal mortality rate was 29 (9). The main referral health facility is SEWA Rural Hospital, which is a 100-bed first-referral unit. There are two PHCs in the study area, with each PHC having a team of two doctors, four ANMs, three male multipurpose health workers, and other support staff. There was one ImTeCHO facilitator and two ERT members. The pilot underwent three stages of delivery of the intervention as described above. ASHAs were to enroll all pregnant women, newborn babies along with their mothers, and young children up to 2 years of age who resided in their respective villages. Subsequently, ASHAs were expected to regularly log in to the ImTeCHO application and complete assigned tasks.

This formative study was done primarily using qualitative methodology. In-depth interviews, focus group discussions (FGDs), and notes from supervisory visits during delivery of the intervention were used. Respondents were purposively selected for the interviews. Six ASHAs were individually interviewed at the community level and nine ASHAs participated in a FGD at SEWA Rural campus. All six visits conducted by the sampled ASHAs on the day of the interview were observed. The two medical officers were individually interviewed at their respective PHCs. Two FGDs were conducted with six ANMs as well. Interviews and FGDs were conducted by a team of researchers, one external and two belonging to SEWA Rural, none of whom were involved with development of the intervention or training of the ASHAs. Informed consent was obtained from all participants, and approval was obtained from the Multi-Institutional Ethics Committee in Mumbai prior to initiation of the study. For analysis, acceptability and feasibility issues were categorized under five major themes, and Weft-QDA statistical software (version 1.0.1) was used for analyzing data (24). Another study was done to field-test all videos that were used as BCC job-aid, the results of which will be reported as a separate publication.

#### Participation with ImTeCHO

A total of 1,100 pregnant women and 1,422 children were registered by the ASHAs. All ASHAs demonstrated enough competencies to get certified to use ImTeCHO. None of the ASHAs stopped using the mobile phone application during the pilot. Two women stopped working as ASHAs during the course of the project for reasons unrelated to the project, and their replacements are now using the ImTeCHO application. There was no reported instance of any beneficiary or ASHA refusing to participate. The average login rate during the study period was 88%; however, the login rate for medical officers was only 17%. During the pilot phase, 10,774 tasks were generated, of which 7,710 (71%) were completed by the ASHAs.

#### Acceptability, feasibility, and usefulness of components

During the interviews, all ASHAs found the ImTeCHO and its components quite acceptable, feasible, and useful. Some of the issues that arose during the pilot are listed here: 1) Some beneficiaries found the videos monotonous after a certain amount of repetition. 2) Scheduling of home visits was appreciated; however, ASHAs expressed the need to add scheduling for services to be provided during VHND as well. 3) None of the ASHAs reported any additional instances of proactive supervision from PHCs through the use of ImTeCHO. On the other hand, interaction and supervision from the facilitators and ERT from SEWA Rural occurred regularly whenever any ASHA seemed to fall behind the ImTeCHO process indicators or diagnosed a complicated case. 4) Limited phone memory occasionally created technology-related issues, with the increasing requirement to store data on the mobile phone. 5) The PHC staff found the ImTeCHO incentive management system rigid and less user-friendly. 6) Higher-level officials expressed the need to add features to facilitate and assess the progress made in enrolling new pregnancies and delivery outcomes.

#### Acceptability and feasibility of the delivery process

Overall, all ASHAs found delivery of the intervention largely acceptable and implementable. The process indicators, available in real-time with the help of mobile-phone technology, were an innovative tool that proved quite useful for monitoring delivery and adherence to the intervention. The interaction between the ASHA and beneficiary was largely structured by the content of the digital form required to be completed. It became clear that the mobile phone application lent a certain degree of formality to the visit. Two ASHAs experienced difficulty in receiving and transferring data because of lack of GPRS in their villages for prolonged period of time. At the outset, there were quite a few instances of ‘false positive’ diagnoses due to ASHAs inadvertently entering the wrong information in the application; however, the proportion of false positive diagnoses sharply declined over the study period to less than 5% at the end.

Quite a few changes were made to the components of the intervention and its delivery; the acceptability and feasibility of these changes were again tested through an iterative process. To reduce false positive diagnoses, the refresher training was modified accordingly and a new screen was introduced to confirm positive findings before finally showing a diagnosis. To overcome limitations of the Java platform, we moved to the Android platform, a move which was made easy thanks to a sharp decline in the cost of Android phones. Processes within the ImTeCHO incentive management system were further simplified to make it more user-friendly. ASHAs found patient-care support provided by the ERT quite valuable; however, creating a new ERT cadre within the government system seems to be an unlikely proposition. It became obvious that patient-care support for most common complications could be provided via phone. Also, the ANMs and medical officer took on more and more of the responsibility for providing patient care. Hence, we decided to remove the ERT cadre and establish a Patient Management Helpline. To deal with the less-than-expected uptake of intervention by medical officers and PHC staff, we introduced additional features that would create value for them and further simplify their tasks. Measures to strengthen monitoring and adherence of PHC staff were introduced by garnering commitment from higher-level officials and introducing simple and key monitoring parameters through the ImTeCHO web application.

## Discussion

This article describes the process of development and evaluation of ImTeCHO, a complex intervention aimed at overcoming key implementation bottlenecks in the ASHA program through the use of mobile phone technology. The intervention was implemented within the primary health system of the government to assess acceptability, feasibility, and usefulness of intervention along with barriers encountered during its delivery. Quite a few lessons learned through the evaluation process helped to further improve the intervention. The final intervention will be now tested in a field trial.

The MRC framework for developing and evaluating a complex intervention was used. Through our experience of working with village-based frontline health workers over the last three decades and a literature review, it was concluded that insufficient support, supervision, and motivation were some of the most important reasons for suboptimal performance of the ASHA program, leading to low coverage of selected proven interventions. To overcome the above problems of implementation, the ImTeCHO intervention was created, consisting of four components as job-aids to ASHAs and PHC staff and drawing upon principles of implementation science and user-centered design to build a platform based on mobile phone technology. The feasibility, acceptability, and usefulness of ImTeCHO were then tested through a pilot study in a population of 45,000, through 45 ASHAs and the staff of two PHCs with active facilitation by SEWA Rural. Based on the findings of the study, the ImTeCHO intervention was modified in following manner: 1) the ERT cadre was dropped and patient-care support provided through a helpline; 2) features were added and the processes simplified to increase uptake among medical officers and PHC staff; 3) the incentive management system was simplified; and 4) the application was moved to the Android platform.

The final model (Table 2) has two sets of components: 1) scheduling, BCC, and diagnosis and patient management job-aid to be largely delivered by ASHAs; and 2) support and supervision job-aid to be delivered primarily through PHC staff, including the medical officers. SEWA Rural will assist though a helpline and cadre of facilitators. The initial preparatory, training, and stabilization stage will last for 2 months, followed by a subsequent maintenance stage. The ImTeCHO intervention will now be tested through a cluster randomized trial, with the PHC being the unit of randomization. The trial will take place in six tribal blocks of Gujarat. Each PHC will be randomized to the intervention arm or the control arm where routine care would continue.

**Table 2:** Final ImTeCHO model

Intervention and delivery components	Specific technology inputs through ImTeCHO	Specific actions/outputs from users to follow technology inputs	Timing of delivery
Scheduling job-aid	The system generates and displays ‘ASHA's Daily schedule/task list’ for each user on mobile or web interface. ASHA's Daily schedule/task list, which is updated daily, has a list of reminder alerts displaying names of beneficiaries with services due and administrative tasks with due dates for each task.	ASHAs log in to the ImTeCHO application every day and complete scheduled tasks on time by visiting beneficiaries at home, along with completing administrative tasks.	Mainly during stabilization and maintenance phase
	The system tracks the status (complete or pending) of each task. The system removes a task from ‘ASHA's Daily schedule/task list’ once the ASHA complete the task. The color of text displaying the task changes to red if task becomes overdue.		
	The system generates and displays a preservice list (at ImTeCHO application at mobile end) of services due to be provided during VHND.^a^	ASHAs mobilize all beneficiaries who are due for services during VHND.	
BCC job-aid	Multimedia (short videos, photos) is embedded within home visit. Having multimedia as first screen during home visit reduces formality of interpersonal relationship.^a^	ASHAs use multimedia and checklist for effective counseling towards BCC during home visits.	Mainly during stabilization and maintenance stage
	Digital form to be completed during home visits contains checklists to assess and address determinants of behavior change of families.		
Diagnosis and patient management job-aid	ImTeCHO point-of-care decision support system provides probable diagnosis through in-built algorithms based on information entered.	ASHA interviews and examines beneficiary and enters information in digital form to screen, identify, risk stratify, and manage complicated cases.	Mainly during stabilization and maintenance stage
	Phone as a tool for ASHAs to facilitate referral. There is a feature to call emergency transportation through application.	ASHA use mobile phone to call emergency transportation.	
	System sends notification alerts via SMS and schedules home visit for PHC medical officer/ANM once complicated case is identified by ASHA.	PHC medical officer and ANM follow up on complicated cases for timely management.	
	The system displays customized patient-management guidelines on mobile phone based on diagnosis.	ASHAs manage complicated cases at home who refuse to get referred to health facility.	
	The system generates tasks to remind ASHAs to make follow-up home visits to complicated cases.	ASHAs ensure adherence to suggested treatment.	
	System sends notification alerts to the helpline representative's (a qualified health provider) dashboard on web interface once a complicated case is identified by ASHA.^a^	ASHAs receive guidance from a qualified provider over phone to manage complicated cases who refuse to get referred to health facility.	
Supervision and support management job-aid	ASHA performance information module at the ImTeCHO mobile and web end will display objective performance parameters for each ASHA in real time.^a^	Supervisory staff use ASHA performance information module during supervisory field visits.	Mainly during stabilization and maintenance stage
	A dashboard for medical officer displaying reports regarding coverage of selected key interventions (e.g. immunizations), list of complicated cases, drug stock outs, pending incentive payments, and recent deaths.	Medical officer and PHC staff use reports to supervise ASHAs, replenish drugs, etc., during monthly PHC meeting.	
	ASHA incentive management system automatically calculates monthly incentives and records their payment.	PHC staff disburses incentives based on ready-made activity report available through the ImTeCHO web.	
	Bulk announcements could be sent to ASHAs to recognize instances of extraordinary effort made for patient care, automated congratulatory messages that appear on mobile if ASHA achieves desired performance standards, etc.	Program management cell regularly uploads announcements. ASHA receives additional performance-based incentives, based on task completion rate from the ImTeCHO program.	
	The system displays relevant information to assist PHC staff with timely and accurate data entry into other MNCH registries of health department.^a^	PHC staff use the ImTeCHO interface to complete data entry into other MNCH registries.	
	Selected performance parameters of medical officers made available for their supervisors at block, district, and state levels through web application and monthly emails.	Higher-level health official supervises medical officers and gives relevant instructions.	
Training, mentoring, and certification	A separate training application that is used to practice and get hands-on experience	The training team uses training application for training (approximately 7 days) of ASHAs. One to two days’ training of PHC staff for use of the ImTeCHO web interface. Focus on reducing effect of using technology on interpersonal communication.^a^	Mainly during training stage
	ASHA activity log on web has detailed information about ASHAs’ use of application.	The training team remotely (instead of in the field) mentors ASHAs through series of phone calls based on their activity logs during first few weeks after training.^a^	
Patient management helpline	Helpline dashboard has information about all complicated cases identified by ASHAs.	A team of qualified health providers operate helpline to contact ASHAs and beneficiaries to provide guidance over the phone for patient management.	Mainly during stabilization and maintenance stage
ImTeCHO facilitator (IF)	The web interface shows process indicators report.	IF supervises ASHAs and conveys feedback during monthly performance-review phone call and additional calls in between if indicated based on process indicators.	Mainly during training and stabilization and maintenance stage
	Helpline for technology support	IF provides technology support in case of any technology/mobile phone/ImTeCHO application-related problems reported by users.	
		IF attends monthly PHC meeting and makes occasional field visits to those ASHAs whose performance parameters within ImTeCHO is consistently poor.	
Program implementation cell atheadquarters	A dashboard to show process indicators, which are tools for monitoring the ImTeCHO intervention Monthly email and SMS to medical officers and higher-level health officials about performance parameters	The cell supervises and supports the ImTeCHO facilitators, program coordinators, and helpline. Coordinates with medical officers and higher level health officials	Throughout the delivery of intervention, including preparatory, training, and maintenance stages
		Intervene in emergency situation	
		Develop and improvise software application with help of IT partner	

a Components that were added or modified based on findings of the pilot.

Even though the use of mHealth interventions is becoming common, the literature on development of such interventions is sparse. One of the important areas that need to be explored is how mHealth can address challenges faced by those working in the MNCH domain, instead of the technology being an end in itself. This article tries to bridge this gap by articulating the gap in current care, how mHealth solutions were developed to address this gap, and evaluation of the solutions. This study, to best of our knowledge, is the first one to document development of a complex intervention involving mHealth in the domains of implementation science and MNCH using methodology prescribed by the MRC.

To conclude, ImTeCHO and its delivery were found to be acceptable, feasible, and useful within the primary health care system and the ASHA program.

## Supplementary Material

Development and formative evaluation of an innovative mHealth intervention for improving coverage of community-based maternal, newborn and child health services in rural areas of India
