# Supplementary material for: Development and formative evaluation of an innovative mHealth intervention for improving coverage of community-based maternal, newborn and child health services in rural areas of India
Source: Glob Health Action. 2015 Feb 16;8:10.3402/gha.v8.26769. doi: 10.3402/gha.v8.26769 (PMC4335194; doi:10.3402/gha.v8.26769)
Supplement: Development and formative evaluation of an innovative mHealth intervention for improving coverage of community-based maternal, newborn and child health services in rural areas of India [file GHA-8-26769-s001.pdf]

Supplementary files

1. Counseling tools, in form of short videos (picture stories) that is being played on mobile phone by frontline workers, is available at http://www.youtube.com/user/sewarural
2. Training tools including introductory training video is available at http://www.youtube.com/watch?v=c8VJ7QjZLo8
3. Project website is www.imtecho.com
